# Supplementary figures and images for: Effects of imatinib on vascular insulin sensitivity and free fatty acid transport in early weight gain
Source: PLoS One. 2021 Jul 2;16(7):e0250442. doi: 10.1371/journal.pone.0250442 (PMC8253421; doi:10.1371/journal.pone.0250442)

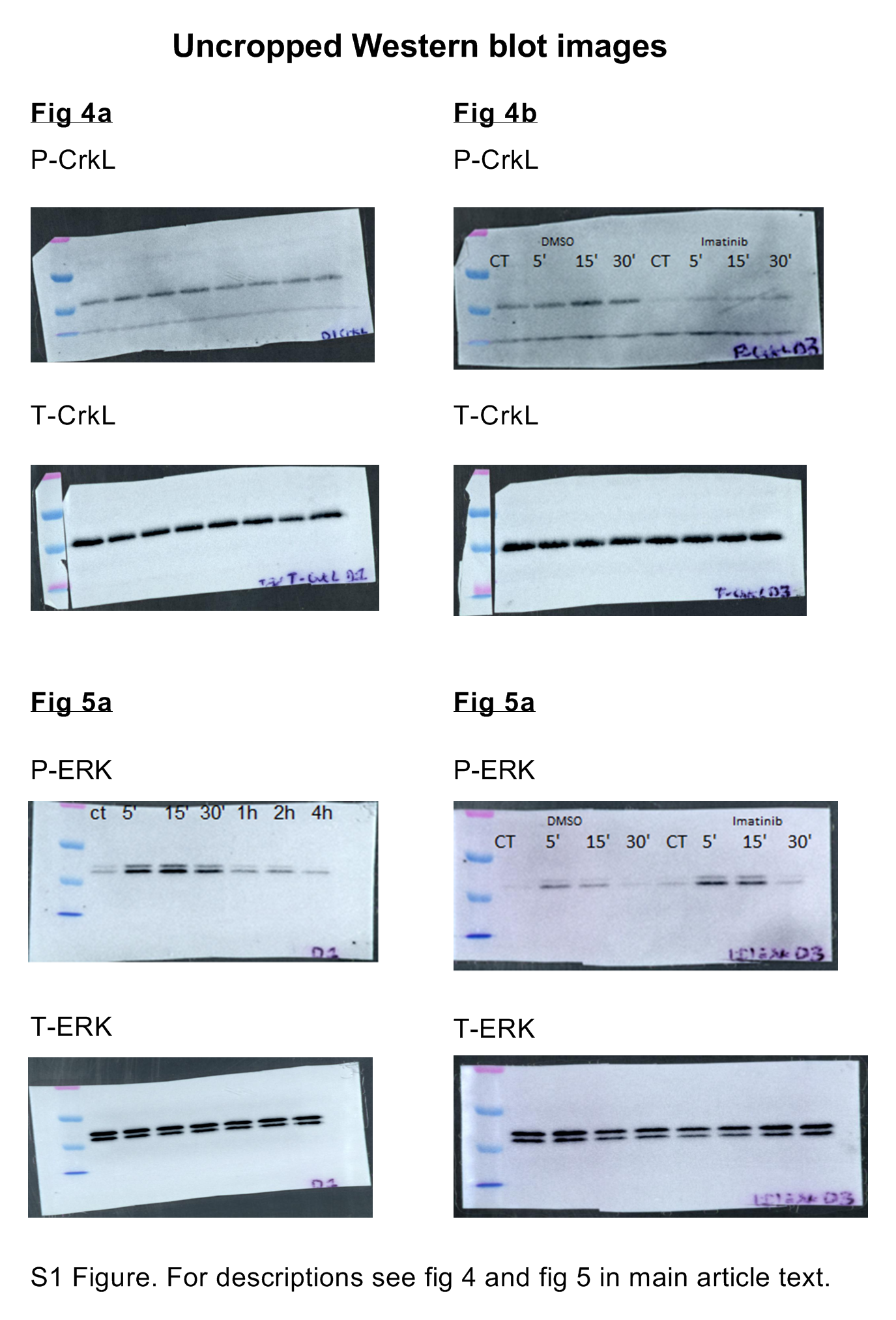

Supplement: S1 Fig — (TIF) [file pone.0250442.s001.tif]
