# Supplementary material for: Effects of imatinib on vascular insulin sensitivity and free fatty acid transport in early weight gain
Source: PLoS One. 2021 Jul 2;16(7):e0250442. doi: 10.1371/journal.pone.0250442 (PMC8253421; doi:10.1371/journal.pone.0250442)
Supplement: Attachment — Submitted filename: Comments.docx [file pone.0250442.s002.docx]

**Reviewer**

**General comments:**

Inconsistent format appears in the manuscript. For example: Page 8 line 206: two-way ANOVA and Page 9 line 235: 2-way ANOVA.

Most of the content in the introduction is about type 2 diabetes, but the research related to this aspect is relatively insufficient. Whether use DB mouse model for further research to investigate the effects of imatinib on metabolic insulin sensitivity?

**Specific comments:**

1. Introduction: The ideas presented (Since VEGF-A and VEGF B stimulate the same receptor，Page 4 lines 92 through 93) are not rigorous. VEGF-A is the prototypical member of the family and a strong angiogenic inducer, predominately by signaling through VEGFR2. VEGF-B binds specifically to VEGFR1 and to the common co-receptor neuropilin-1 (NRP1) to regulate lipid metabolism.
2. Methods: Page 4 line 111: Most dietary lipids are long-chain FAs (LCFAs; >12 carbons), whether the product of C1-BODIPY®500/510 C12 is your best choice to study endothelial FA transport in vitro?
3. Results:
a. An expression error of ImatWD mice in saline treated mice appear Page 9 line 227. Line 234: Weight-gaining effect was rescued by imatinib treatment (P=0.006), why express P-values only one *?
b. Figure 2 need more evidence to examine the effects of imatinib on metabolic insulin sensitivity (for example: ITT). Why are there decreasing trends of the fasting blood glucose level in WD mice comparing with chow diets with or without imatinib treatment?
c. Figure 4: the same expression of P-values* in figure 4. Page 11 line 284: there is an expression error of (P>0.9).
d. Figure 5: Writing errors of VEGF-B (100 ng/ml) in figure 5 A-D. Whether VEGFB and imatinib treat endothelial cells together can better explain imatinib affects the VEGF-B mediated phosphorylation of CrkL.
a. Figure 7: More experiments is needed to improve VEGF-B stimulation caused a increase in the uptake of fatty acids corresponding with increased phosphorylation of Arg substrate CrkL at Tyr207. There need an additional treated group of VEGFB、VEGFB antibody and imatinib to further improve the effect of imatinib on VEGFB mediated endothelial FA uptake.
4. Discussion:
1. Page 14 line 382: The available data do not support this view.
2. As mentioned in the discussion (Page 16 lines 414 through 417), the treatment time in mouse model may be too short and could be extended.
3. Insufficient data cannot explain the opinion that Abl kinases mediate VEGF-B signaling without affecting VEGFB-mediated uptake of fatty acids.
