# Supplementary material for: Effects of imatinib on vascular insulin sensitivity and free fatty acid transport in early weight gain
Source: PLoS One. 2021 Jul 2;16(7):e0250442. doi: 10.1371/journal.pone.0250442 (PMC8253421; doi:10.1371/journal.pone.0250442)
Supplement: Attachment — Submitted filename: Response to reviewers PONE-D-19-35443.docx [file pone.0250442.s003.docx]

**Response to reviewer #1:**

Thank you for your time and careful review of our paper. We will answer to each of your points below:

**Major comment 1:** “If body weight is not equivalent, a change in insulin sensitivity and glucose levels is expected. A beneficial finding is thus moot. It seems that the authors are celebrating this finding as positive, when really the mouse exhibits a lipodystrophy-like phenotype. Thus, despite being lower in mass, glucose levels are equivalent (close to being higher even on chow) and insulin sensitivity is barely different. What are the actual masses of these mice (not % change)? Body composition? That muscle takes on equivalent lipid, despite leaner mice, is of metabolic concern.”

Reply: We specifically chose a model for early weight gain to study the effect of imatinib on the earliest steps in the pathogenesis of type 2 diabetes and its cardiovascular complications. The lack of a change in whole-body insulin sensitivity and fasting glucose levels is consistent with other data of our recent human study [1] and is likely explained by a compensatory increase in adipose tissue glucose uptake. Interestingly, imatinib seems to have a protective effect on the body-weight increasing effect of the WD without affecting food intake. For clarification we have now, as suggested by the reviewer, included the final body weight of the mice. We did not obtain information on body composition during the experiments, as we do not have a DXA scanner for mice or similar technology in our animal facility. Most interesting about this model of early weight gain is that we already see an effect of the WD on resistance arteries in vivo before there is an effect on metabolic insulin sensitivity, both in this study and in our human study on early weight gain [1]. Therefore we have included a reference to our group’s new study performed in humans in the discussion section and elaborated on this result to clarify its clinical significance. This is interesting because it shows us how imatinib affects the development of type 2 diabetes and where to look for potential new therapeutic targets.

**Major comment 2:** “In vitro, wouldn't inhibited phosphorylation of downstream VEGFR2 targets be expected as imatinib is a broad-spectrum tyrosine kinase inhibitor? Data on pErk or pCrkl (are) therefore not very interesting. Why would the authors expect differently? The mechanism of imatinib action must be described in the intro and explained relative to the results.”

**Reply**: We have added the mechanism of action of imatinib in the introduction and have also explained it relative to results in the discussion with following text:

“We found that VEGF-B enhances the phosphorylation of CrkL, a downstream target of Abl kinases c-Abl and Arg. This is a new insight, because until now only barrier disruptive agents are known to stimulate Abl kinases [21]. As VEGF-B is known to activate only VEGFR1, this could mean that VEGF-B directly activates Abl kinases through VEGFR1. Although VEGF-B increased phosphorylation of CrkL (downstream target of Arg/c-Abl kinases), no effect of imatinib was observed on VEGF-B induced phosphorylation of ERK. This indicates that ERK1/2 and Abl kinase are independently activated by binding of VEGF-B to VEGFR1, and that imatinib selectively inhibits Abl kinase activation.

By demonstrating that 1) VEGF-B enhances FA uptake in endothelial cells, 2) imatinib inhibits VEGF-B-induced Abl kinase activation, and 3) imatinib does not affect FA uptake in endothelial cells, our study indicates that VEGF-B mediated FA uptake is independent of Abl kinase.”

**Major comment 3:** “The most significant finding is the inability of imatinib to impact VEGF-B mediated FA uptake. This is very interesting and the authors should focus on discussing this mechanism and how it fits into past VEGF-B FA transporter work, VEGFR2 signaling, and the 'controversy' of whether/how VEGFB does this. If imatinib inhibits tyrosine kinases, if there some other pathway?”

**Reply**: We have explained the mechanism of action of imatinib and VEGF-B signalling relative to the results in discussion. “We found that VEGF-B enhances the phosphorylation of CrkL, a downstream target of Abl kinases c-Abl and Arg. This is a new insight, because until now only barrier disruptive agents are known to stimulate Abl kinases [21]. As VEGF-B is known to activate only VEGFR1, this could mean that VEGF-B directly activates Abl kinases through VEGFR1. Although VEGF-B increased phosphorylation of CrkL (downstream target of Arg/c-Abl kinases), no effect of imatinib was observed on VEGF-B induced phosphorylation of ERK. This indicates that ERK1/2 and Abl kinase are independently activated by binding of VEGF-B to VEGFR1, and that imatinib selectively inhibits Abl kinase activation.

By demonstrating that 1) VEGF-B enhances FA uptake in endothelial cells, 2) imatinib inhibits VEGF-B-induced Abl kinase activation, and 3) imatinib does not affect FA uptake in endothelial cells, our study indicates that VEGF-B mediated FA uptake is independent of Abl kinase.”

**Minor comment 1:** “Several of the figure panels should probably be combined. Currently 1+2+3, though this may change if the authors provide body composition or muscle pAkt.”

**Reply**: We have combined figures 1 and 2 into a single figure and have added the total body weight of the mice to figure 1. What was formerly figure 3 is now figure 2. We decided not to combine that figure with the others, because it shows the results concerning a different mechanism with a different methodology even though they were obtained during the same experiment. We want to show the vascular effects of insulin and imatinib separately from the metabolic effects. Although we agree with the reviewer that body composition would have added valuable insight, we did not have equipment to analyze body composition or enough muscle tissue for western blots of pAkt. We have, however, added data on expression of IRS1 and Glut4, other mediators of insulin signalling (figure 3). We feel that these data have added mechanistic insight and thank the reviewer for her/his suggestion.

**Minor comment 2:** “the physiology of vessel dilation is commendable, but multiple mechanisms could be at play (1) if truly more insulin sensitive, this result is expected (2) discussion of the VEGFR2-nitric oxide axis?”

Reply: In our experiments we have used insulin-mediated vasodilation of resistance arteries in vivo as a measure for (micro)vascular effects of insulin, i.e. vascular insulin sensitivity [4]. We used a hyperinsulinemic-euglycemic clamp in combination with intravital microscopy to view the effect of imatinib on the reaction of the vessel diameter to insulin infusion. While imatinib may influence VEGFR2 and its nitric oxide axis to influence vessel dilation, a change in baseline vessel diameter would be expected. We did not observe such a baseline diameter change, and we specifically examined the effect of insulin on vessel diameter. The effect of imatinib we observed specifically concerned insulin-mediated vasodilation, not VEGFR2-mediated vasodilation. We have therefore not changed the manuscript according to your comment and hope this explanation is satisfactory.

**Minor comment 3:** “use of standard deviations would better illustrate why the results that are close but not significant are not actually signification.”

Reply: We have now used standard deviations instead of standard error of means for all results.

**Minor comment 4:** “headings in the conclusion are required?”

Reply: We have inserted the heading for the conclusion.

**Minor comment 5:** “line 379. to say that it is protective is not valid if no tissue pAkt response or other factors are examined? permeability?”

Reply: The factor we examined was insulin-induced vasodilation of a resistance artery and imatinib showed a protective effect against WD-feeding for this factor. Previously, Rask-Madsen et al. have shown that normal vascular insulin signalling protect against atherosclerosis [5]. As a result of your comment we have changed the sentence as follows to clarify this in the manuscript: “Here, we show that imatinib protects the endothelium from the negative effects of a WD on insulin-induced vasodilation of resistance arteries, one of the important endothelial functions that depends on vascular insulin sensitivity.”

**Minor comment 6:** “line 434. Only uptake of FA is shown, not permeability.”

Reply: The reviewer is correct, we have shown uptake of fatty acids in this paper but not transendothelial transport. Unfortunately, we were unable to conduct experiments regarding endothelial permeability. A main reason for this is that endothelial FA uptake, an important step in FA transport, can be monitored using more sensitive microscopy techniques. We have also adjusted this in the introduction part of the article with the following text: “Furthermore, we used an in vitro model of cultured human umbilical vein endothelial cells (HUVECS), to investigate (a) the effect of imatinib on endothelial FA-uptake with and without VEGF-B stimulation”.

**Minor comment 7:** “no discussion of Robciuc 2016 on VEGFB mice being leaner is included.”

Reply: We have now included the elegant study of Robciuc in our discussion, and have discussed their finding of leaner mice after VEGF-B enhancement. “We found that VEGF-B causes a time-dependent increase in the uptake of fatty acids in HUVECS. This is in contrast with the study conducted by Robciuc et al. [44] who found that enhanced VEGF-B/VEGR1 signaling improves glucose metabolism in obese mice through increased VEGF-A activity, angiogenesis and thermogenesis in adipose tissue, which explained their lower body weight after high-fat diet. As such processes (angiogenesis and thermogenesis) are absent in our in vitro experimental set-up, our in vitro data support a direct effect of VEGF-B on fatty acid uptake in endothelial cells. In muscle, the normal VEGF-B and increased VEGFR1 expression of Western diet-fed mice suggest that lipid accumulation in muscle is mediated by increased VEGFR1 signaling rather than increased VEGF-B expression.”

**Minor comment 8:** “final limitations: the authors mentions several but fail to address some of the criticisms raised by this reviewer that should seem obvious?”

Reply: We have expanded the limitations section: we used a specific anesthesia mixture in this experiment, because of normal insulin sensitivity, however some of the mice experienced breathing difficulties during anesthesia and died prior to finishing the entire experiment. This resulted in missing data.

**Response to reviewer #2:**

Thank you for your comments on our manuscript. We have answered each of your comments below.

**General comment 1:** “Inconsistent format appears in the manuscript. For example: Page 8 line 206: two-way ANOVA and Page 9 line 235: 2-way ANOVA.”

Reply: We have changed “2-way ANOVA” into “two-way ANOVA” throughout the manuscript.

**General comment 2:** “Most of the content in the introduction is about type 2 diabetes, but the research related to this aspect is relatively insufficient. Whether use DB mouse model for further research to investigate the effects of imatinib on metabolic insulin sensitivity?”

**Reply**: We have included the following explanation in the introduction in order to clarify our choice for this mouse model: “We chose a mouse model for early weight gain specifically to study the effect of imatinib on the early pathogenesis of type 2 diabetes, since imatinib has been shown to affect endothelial function, and endothelial dysfunction is an essential part of the early pathogenesis of the disease.” We have also included the following sentence in the discussion: “In future research it would also be very interesting to see the effects on later stages of the disease with db/db mice for instance, especially to study the effect on metabolic insulin sensitivity in more depth which we could not do in this study.”

**Specific comment 1:** “Introduction: The ideas presented (Since VEGF-A and VEGF-B stimulate the same receptor，Page 4 lines 92 through 93) are not rigorous. VEGF-A is the prototypical member of the family and a strong angiogenic inducer, predominately by signalling through VEGFR2. VEGF-B binds specifically to VEGFR1 and to the common co-receptor neuropilin-1 (NRP1) to regulate lipid metabolism.”

**Reply**: VEGF-A stimulates both VEGFR-1 and VEGFR-2 receptors. VEGF-A has a 10x higher affinity for VEFGR1 than VEGFR2, yet the activation of VEGFR-2 by VEGF-A mediates its angiogenic properties. VEGFR-1 acts as a decoy receptor for VEGF-A. VEGF-B binds to and activates only to VEGFR-1 [3,6]. Both VEGFR1 and VEGFR2 have a common co-receptor, Neuropilin 1. The binding of VEGF-B to VEGFR-1 plays an important role in angiogenesis, inflammation and fatty acids [7]. According to the reviewer’s suggestion we have elaborated on this topic in the Introduction: “Arg is stimulated by endothelial barrier-disruptive agents such as VEGF-A and histamine [21]. VEGF-A stimulates both VEGFR1 and VEGFR2 receptors. VEGF-A has 10x more affinity for VEFGR-1 than VEGFR-2. However, the stimulation of VEGF-A with VEGFR2 is responsible for its angiogenic properties. VEGFR1 acts as a decoy receptor for VEGF-A [28]. VEGF-B binds to and activate only VEGFR1 [29]. Both VEGFR1 and VEGFR2 have a common co-receptor known as Neuropilin 1. Since VEGF-A and VEGF-B stimulate the same receptor VEGFR1 [12], it might be possible that VEGF-B also stimulates Abl kinases to increase the transport of fatty acids.”

**Specific comment 2:** “Methods: Page 4 line 111: Most dietary lipids are long-chain FAs (LCFAs; >12 carbons), whether the product of C1-BODIPY®500/510 C12 is your best choice to study endothelial FA transport in vitro?”

Reply: We feel that this is a good model, as C1-BODIPY®500/510 C12 has been used in several different studies. Hagberg et al, 2010 used C1-BODIPY®500/510 C12 for their experiments to demonstrate the uptake of long chain fatty acids induced by VEGF-B. As this paper is used as one of the references in our paper, we have used the same type of fatty acids as used in the Hagberg paper.

In addition, Nie et al., 2012 also used C1-BODIPY®500/510 C12 to study the uptake of hepatic fatty acid uptake [8].

Verlamov et al., 2010 also used these bodipy fatty acids to study the insulin-regulated fatty acid uptake in adipocytes [9].

**Specific comment 3a:** An expression error of ImatWD mice in saline treated mice appear Page 9 line 227. Line 234: Weight-gaining effect was rescued by imatinib treatment (P=0.006), why express P-values only one *?”

Reply: In this line have changed “*P<0.05” according to your comment into “**P<0.01.”

**Specific comment 3b:** “Figure 2 need more evidence to examine the effects of imatinib on metabolic insulin sensitivity (for example: ITT). Why are there decreasing trends of the fasting blood glucose level in WD mice comparing with chow diets with or without imatinib treatment?”

Reply: ITT is indeed a useful way to measure metabolic insulin sensitivity. We chose to use the hyperinsulinemic-euglycemic clamp, because it is considered the gold standard for measuring peripheral insulin sensitivity. With regards to the blood glucose levels, we decided not to discuss the decreasing trend in the results of the fasting blood glucose levels, because is not a significant result. One reason why fasting blood glucose levels might be lower in the WD-fed mice might be that increased angiogenesis in adipose tissue when exposed to high-caloric diet causes an increase in glucose uptake by these fatty tissues and thus a lowering of the blood glucose levels. This however is also speculation and we therefore also decided not to discuss this in the article. We have, as suggested by the reviewers, elaborated on the lack of an effect of the WD on metabolic insulin sensitivity and glucose levels. We have now included a reference to a human study done by our group to the discussion section to show that in the early stages of diet-induced weight gain metabolic insulin sensitivity is not yet affected, but vascular insulin sensitivity is. This is in fact what our results of the early effects of a high-fat diet show and we hope that this elaboration is satisfactory to explain that.

**Specific comment 3c:** “Figure 4: the same expression of P-values* in figure 4. Page 11 line 284: there is an expression error of (P>0.9).”

**Reply**: In Figure 3 (formerly Figure 4) we have changed the * for P<0.05 to ** for P<0.01 since the one significant P-value in this figure is P=0.009.

**Specific comment 3d:** “Figure 5: Writing errors of VEGF-B (100 ng/ml) in figure 5 A-D. Whether VEGFB and imatinib treat endothelial cells together can better explain imatinib affects the VEGF-B mediated phosphorylation of CrkL.”

**Reply**: In Figure 4 (formerly Figure 5) we have changed 100ng into 100ng/ml, since that is what has been used. As to the other part of the reviewers comment we are sorry to say that we are unsure what is asked or suggested here. Therefore we were sadly unable to reply to it properly.

**Specific comment 3e:** “Figure 7: More experiments are needed to improve VEGF-B stimulation caused an increase in the uptake of fatty acids corresponding with increased phosphorylation of Arg substrate CrkL at Tyr207. There need an additional treated group of VEGFB、VEGFB antibody and imatinib to further improve the effect of imatinib on VEGFB mediated endothelial FA uptake.”

**Reply**: It was unclear in figure 6 (formerly figure 7) that we had a group treated with imatinib but without VEGF-B, since we used plus-signs and minus-signs to show whether we had treated the cells with VEGF-B. This could result in confusion. Therefore we have changed this in the figure to “with/without VEGF-B”. Since we used a group with imatinib, but without VEGF, an additional group treated with VEGFB, VEGFB antibody, and imatinib would be redundant. VEGFB antibody would cancel the effect of VEGFB, giving the same effect as no VEGFB at all, resulting in the same data as the group we have currently.

**Specific comment 4.1:** “Page 14 line 382: The available data do not support this view.”

**Reply**: We have used insulin-induced vasodilation of resistance arteries as a measure for vascular insulin sensitivity. We have changed the line that you refer to in order to clarify this and have clarified this aspect in the text beneath this header as well.

**Specific comment 4.2:** “As mentioned in the discussion (Page 16 lines 414 through 417), the treatment time in mouse model may be too short and could be extended.”

**Reply**: It is true that our treatment period has benefits and limitations. In response to the reviewers’ comments we have elaborated on our choice for this treatment time in the introduction by stating: “We chose a mouse model for early weight gain specifically to study the effect of imatinib on the early pathogenesis of type 2 diabetes, since imatinib has been shown to affect endothelial function, and endothelial dysfunction is an essential part of the early pathogenesis of the disease.” We agree with the reviewer that an extended treatment time would be a very interesting addition to this research. However, a short intervention avoids confounding effects of obesity, hyperinsulinemia, hypertension and hyperglycemia that would complicate interpretation of data. An extended treatment time would give us insights into the effect of imatinib on the disease in a further stage of development. In our current research we chose to focus on the early stages of the disease for reasons mentioned above. As now stated in the ‘strengths and limitations’ section of our discussion, future research into the further stages of the disease would indeed be very interesting: “In future research it would also be very interesting to see the effects of imatinib on later stages of the disease using db/db mice, especially to study the effect on metabolic insulin sensitivity in more depth which we could not do in this study.”

**Specific comment 4.3:** “Insufficient data cannot explain the opinion that Abl kinases mediate VEGF-B signaling without affecting VEGFB-mediated uptake of fatty acids.”

Reply: We have shown that VEGF-B enhances the phosphorylation of CrkL (downstream target of Arg (Abl related gene) and ERK. VEGF-B also induced a time dependent increase in the uptake of fatty acids. Imatinib inhibited the VEGF-B-mediated phosphorylation of CrkL, but caused no effect on the VEGF-B mediated phosphorylation of ERK. Furthermore, we saw no effect of imatinib on VEGF-B mediated uptake of fatty acids. Imatinib predominantly exerts its endothelial effects via inhibiting Abl kinases. Our data could mean that VEGF-B stimulates VEGFR1 for its effect on the phosphorylation of Arg, since VEGF-B specifically binds to VEGFR1. The inability of imatinib to affect the VEGF-B mediated fatty acid uptake could mean that VEGF-B mediated fatty acid uptake is independent of Abl kinase signaling. We have discussed the relationship between Arg and fatty acid uptake on p.18/19 of the manuscript.

1.	Emanuel AL, Meijer RI, Woerdeman J, van Raalte DH, Diamant M, Kramer MHH, et al. Effects of a Hypercaloric and Hypocaloric Diet on Insulin-Induced Microvascular Recruitment, Glucose Uptake, and Lipolysis in Healthy Lean Men. Arterioscler Thromb Vasc Biol. 2020;40: 1695–1704. doi:10.1161/ATVBAHA.120.314129

2.	Almalki SG, Agrawal DK. ERK signaling is required for VEGF-A/VEGFR2-induced differentiation of porcine adipose-derived mesenchymal stem cells into endothelial cells. Stem Cell Res Ther. 2017;8: 113. doi:10.1186/s13287-017-0568-4

3.	Robciuc MR, Kivelä R, Williams IM, de Boer JF, van Dijk TH, Elamaa H, et al. VEGFB/VEGFR1-Induced Expansion of Adipose Vasculature Counteracts Obesity and Related Metabolic Complications. Cell Metab. 2016;23: 712–724. doi:10.1016/j.cmet.2016.03.004

4.	Turaihi AH, van Poelgeest EM, van Hinsbergh VWM, Serné EH, Smulders YM, Eringa EC. Combined Intravital Microscopy and Contrast-enhanced Ultrasonography of the Mouse Hindlimb to Study Insulin-induced Vasodilation and Muscle Perfusion. 2017; e54912. doi:10.3791/54912

5.	Rask-Madsen C, Li Q, Freund B, Feather D, Abramov R, Wu I-H, et al. Loss of Insulin Signaling in Vascular Endothelial Cells Accelerates Atherosclerosis in Apolipoprotein E Null Mice. Cell Metab. 2010;11: 379–389. doi:10.1016/J.CMET.2010.03.013

6.	Lal N, Puri K, Rodrigues B. Vascular Endothelial Growth Factor B and Its Signaling. Front Cardiovasc Med. 2018;5: 39. doi:10.3389/fcvm.2018.00039

7.	Ceci C, Atzori MG, Lacal PM, Graziani G. Role of VEGFs/VEGFR-1 Signaling and its Inhibition in Modulating Tumor Invasion: Experimental Evidence in Different Metastatic Cancer Models. Int J Mol Sci. 2020;21: 1388. doi:10.3390/ijms21041388

8.	Nie B, Park HM, Kazantzis M, Lin M, Henkin A, Ng S, et al. Specific bile acids inhibit hepatic fatty acid uptake in mice. Hepatology. 2012;56: 1300–1310. doi:10.1002/hep.25797

9.	Varlamov O, Somwar R, Cornea A, Kievit P, Grove KL, Roberts Jr CT. Single-cell analysis of insulin-regulated fatty acid uptake in adipocytes. Am J Physiol Endocrinol Metab. 2010/06/22. 2010;299: E486–E496. doi:10.1152/ajpendo.00330.2010
