# Supplementary material for: Effects of imatinib on vascular insulin sensitivity and free fatty acid transport in early weight gain
Source: PLoS One. 2021 Jul 2;16(7):e0250442. doi: 10.1371/journal.pone.0250442 (PMC8253421; doi:10.1371/journal.pone.0250442)
Supplement: Attachment — Submitted filename: Response to reviewers 2 PONE-D-1 9-35443.docx [file pone.0250442.s004.docx]

**Response to reviewer:**

Thank you for your time and careful review of our paper. We will answer to each of your points below:

**Comment #1:** “The authors have provided some re-writing and edits to help improve the manuscript, but have failed to addressed several concerns due to these requiring further experimentation. These concerns could be addressed by discussing them, rather than merely arguing back to the reviewer that they are unnecessary. Much of this stems from the confusion of the authors trying to integrate imatinib and VEGF-B signaling together, yet not successfully doing either well in of itself. The concluding paragraph of the introduction states that "aim of our study was to investigate the effect of imatinib on endothelial fatty acid uptake and on vascular insulin sensitivity through insulin-mediated vasodilation of resistance arteries". By bringing VEGFB into this, and the conflicting reports of its role in FA uptake (Dijkstra 2014)(or even its intracellular signaling), the message is muddy.

Perhaps VEGFB in the intro and throughout could be downplayed and only introduced as a means to elicit a change in FA uptake? The extensive discussion of this factor makes the reviewer read thinking that the purpose of imatinib is to counter VEGFB throughout... but I do not think that is the authors' purpose?”

**Reply**: The reviewer’s point is well taken, the relationship between insulin’s effects and VEGF-B was indeed not very clear in the introduction. In response we have explained the relationship between cellular fatty acid accumulation and insulin-induced vasodilation in the Introduction (p.3, ll.67-72) as follows:

“Increased levels of FFAs directly impair insulin mediated vasodilation^46^ and trigger low-grade inflammation, which enhances endothelial permeability [23]. One of the potential regulators of fatty acids uptake is vascular endothelial growth factor-B (VEGF-B), which has been proposed to stimulate transendothelial transport (TET) of lipids [5–7]. Although regulation of fatty acid transport and blood flow are key features of endothelial dysfunction in type 2 diabetes, few therapeutic agents improve these processes[6].”

Ref. 21 shows a direct interaction of fatty acids within endothelial cells and insulin’s effects in muscle resistance arteries, linking the two parts of our study.

We agree that the part of the discussion on VEGF-B was a bit extensive and have condensed it following the reviewer’s suggestion.

**Comment #2:** "aim of our study was to investigate the effect of imatinib... on vascular insulin sensitivity through insulin-mediated vasodilation of resistance arteries" This study is commendable, but it is not clear that the experiment is achieving this aim. The aim would be on isolated vessels independent of the mouse's metabolic state, but these are in situ in mice with preserved insulin sensitivity, so the result is less interesting than a direct competition. Would imatinib in the bath of an isolated vessel from an insulin-resistant mouse, or if in situ, direct application to the vessel region examined, elicit the same response? Else this is just further confirmation, like the other metabolic data, that, yes, imatinib-treated animals are healthier. A minor point along those lines is that the authors should not use 'improve' or 'increase' but rather the vasodilatory response is 'preserved' in mice receiving imatinib since this is not a therapeutic application.”

**Reply**: In response to the reviewers observation we have made revisions to the article where it describes this particular aim and experiment, in the introduction, results, and discussion section. We indeed should not have stated that our study directly aims to show an effect of imatinib on vascular insulin sensitivity through insulin mediated vasodilation of resistance arteries, instead our study shows how imatinib preserves insulin-mediated vasodilation of resistance arteries in vivo in mice fed a Western diet. While insulin-mediated vasodilation of resistance arteries is a defining characteristic of normal vascular effects of insulin, it is certainly not the only one and therefore the reviewer is correct in stating that our aim should have been more specific. As the reviewer also rightfully states it would indeed be of added value to investigate whether imatinib elicits this effect directly in an ex-vivo setting to see whether this effect of imatinib in vivo was directly or through some other pathway that is not present in an ex-vivo setting. We have included this suggestion in our discussion section as an interesting part of follow-up research, but answering this question would require a new study on interaction of imatinib with fatty acid impairment of insulin-induced vasodilation of isolated muscle resistance arteries. Thirdly we have, per suggestion of the reviewer, corrected the sentences that stated that the vasodilatory response wat improved and now used the word “preserved” which is indeed more apt.
